# Source of Early Regenerating Axons in Lamprey Spinal Cord Revealed by Wholemount Optical Clearing with BABB

**DOI:** 10.3390/cells9112427

**Published:** 2020-11-06

**Authors:** Guixin Zhang, William Rodemer, Isabelle Sinitsa, Jianli Hu, Michael E. Selzer

**Affiliations:** 1 Shriners Hospitals Pediatric Research Center (Center for Neural Repair and Rehabilitation), Philadelphia, PA 19140, USA; kzhang59@temple.edu (G.Z.); william.rodemer@gmail.com (W.R.); jianli.hu@temple.edu (J.H.); 2 College of Science and Technology, Temple University, Philadelphia, PA 19122, USA; isabellesinitsa@temple.edu; 3 Department of Neurology, the Lewis Katz School of Medicine at Temple University, 3500 North Broad Street, Philadelphia, PA 19140, USA

**Keywords:** lamprey, axonal regeneration, BABB, wholemount, anterograde tracing, retrograde tracing

## Abstract

Many studies of axon regeneration in the lamprey focus on 18 pairs of large identified reticulospinal (RS) neurons, whose regenerative abilities have been individually quantified. Their axons retract during the first 2 weeks after transection (TX), and many grow back to the site of injury by 4 weeks. However, locomotor movements begin before 4 weeks and the lesion is invaded by axons as early as 2 weeks post-TX. The origins of these early regenerating axons are unknown. Their identification could be facilitated by studies in central nervous system (CNS) wholemounts, particularly if spatial resolution and examination by confocal microscopy were not limited by light scattering. We have used benzyl alcohol/benzyl benzoate (BABB) clearing to enhance the resolution of neuronal perikarya and regenerated axons by confocal microscopy in lamprey CNS wholemounts, and to assess axon regeneration by retrograde and anterograde labeling with fluorescent dye applied to a second TX caudal or rostral to the original lesion, respectively. We found that over 50% of the early regenerating axons belonged to small neurons in the brainstem. Some propriospinal neurons located close to the TX also contributed to early regeneration. The number of early regenerating propriospinal neurons decreased with distance from the original lesion. Descending axons from the brainstem were labeled anterogradely by application of tracer to a second TX close to the spinal–medullary junction. This limited contamination of the data by regenerating spinal axons whose cell bodies are located rostral or caudal to the TX and confirmed the regeneration of many small RS axons as early as 2 weeks post-TX. Compared with the behavior of axotomized giant axons, the early regenerating axons were of small caliber and showed little retraction, probably because they resealed rapidly after injury.

## 1. Introduction

The lamprey central nervous system (CNS) has been used extensively to study the mechanisms of axon regeneration after spinal cord (SC) injury, in part because it has identified neurons and neuron types that can be imaged in vivo. The lamprey’s normal symmetrical body locomotion is controlled by central pattern generators in the SC, which are activated by reticulospinal (RS) projections from brainstem [1]. Because these connections are lost after SC transection (TX), the body below the TX site becomes paralyzed. To date, 18 pairs of medium to large RS neurons have been individually identified in the lamprey brainstem, and their regenerative probabilities determined [2,3,4,5,6]. Studies of these neurons have suggested that their axons retract during the first 2 weeks after TX, then grow back toward the TX site. They reach the glial “scar” by 3 weeks, enter the lesion site by 4 weeks, and regenerate into the caudal stump thereafter [7,8,9]. It is therefore surprising that lampreys begin to recover locomotion within 2–4 weeks [7,10,11,12], albeit imperfectly coordinated. Physiological studies showed that, although immediately after SC TX, locomotor muscle activity can be recorded only from segments rostral to the lesion, the activity can already be recorded from segments just caudal to the TX by 2 weeks post-TX; and with increasing recovery times, at progressively more caudal levels [13]. Immunohistochemistry (IHC) studies show that neurofilament-containing neuronal processes invade the “scar” by 2 weeks [14], but the origins of these early regenerating axons are unknown. The difficulty in identifying the sources of these axons stems from a combination of factors. The early regenerating axons are of small caliber and follow less straight paths than control axons, making it very awkward to trace them in serial sections. It would be much more effective if they could be imaged in wholemount preparations, but particularly at early times post-TX, the area of injury presents high background staining and poor visibility due to autofluorescence, invasion of the injury by cells from the blood, and connective tissue on the surface of the scar, which is too fragile to strip of extraneous material. In the present paper, we reexamine the time course of regeneration, using tissue clearing, retrograde and anterograde fluorescent labeling and wholemount imaging, to better view regenerating neurites and identify the sources of early regenerating axons.

Wholemounts of lamprey brain and SC have been studied for many years to elucidate the structure, organization and molecular properties of central neurons, in combination with conventional histology [14], intracellular tracer injection [3,5,8,15], retrograde tracing [2,16,17], IHC [2,18,19,20,21] and in situ hybridization (ISH) [22,23]. A special advantage of wholemounts in the lamprey CNS is the ability to map individual RS neurons, which can be readily identified by their size, shape and spatial relationship to each other. If performed using serial histological sections, these studies would be much more time-consuming, technically difficult, and less accurate. We previously used this mapping advantage to correlate expression of specific genes with the regenerative probabilities of individual neurons. These studies can require double or even triple labeling to colocalize expression of specific genes and to pair IHC with ISH and even caspase activation [19]. However, the thickness and light scattering of wholemount brain and SC can make it challenging to image labeled structures deep within a tissue by conventional bright field or widefield fluorescence microscopy. Further improvements in the resolution of deep structures will require more effective clearing of tissue than has been possible to date. For that reason, we tried an old clearing agent BABB (1 part benzyl alcohol and 2 parts benzyl benzoate) that has been used as an optical clearing agent for decades and provided excellent visualization of the adult and embryonic mouse brain and fruit fly central ganglia [24,25], among numerous other tissues and species [25,26,27,28,29,30]. It works fast and is user friendly, inexpensive and compatible with fluorescent labeling. Its use fell out of favor because it is a toxic solvent, but we have been able to take advantage of its benefits by adapting the BABB protocol to develop a simple, efficient and safe clearing method for imaging wholemounts of lamprey brainstem and SC by either conventional widefield or confocal microscopy.

Numerous previous reports have evaluated axon regeneration of RS neurons after TX by applying an enzyme, horseradish peroxidase (HRP), or a fluorescent dye to a second TX 5–10 mm caudal to the initial TX. These procedures retrogradely label RS neurons whose axons have regenerated as far as the second TX [2,6,31]. However, because most RS neurons regenerate only short distances [3,7,8,32,33], many neurons with regenerated axons will not be labeled, even though they may have formed functioning synapses onto neurons distal to the TX [34,35,36]. Anterograde labeling by intracellular or intra-axonal tracer injection does not have this limitation, but the large volume of many axons can sufficiently dilute the micro-injected tracer that it is not visible all the way to the regenerating axon tip. An alternate approach previously explored in lampreys is to anterogradely label regenerated axons by introducing tracer through a second TX rostral to the original lesion [37,38]. Although this method does not allow identification of the neurons that give rise to the regenerated axons, it has the potential to yield a great deal of important information concerning the sizes and positions of the descending regenerated projections, the distances they regenerate, and the effects of therapeutic interventions. When fluorescent tracer was applied to a re-TX of the original lesion at 2 weeks, or a few mm caudal to the lesion at 2–6 weeks post-TX, to label regenerated neurons by retrograde transport, we confirmed that anterograde labeling of the SC from a second TX ≥ 5 mm rostral to the original lesion labeled RS axons almost exclusively, and introduced only very limited contamination by axons belonging to SC neurons.

## 2. Materials and Methods

### 2.1. Animals and Spinal Cord Transection

Larval sea lampreys (*Petromyzon marinus*), 9–12 cm in length (3–4 years old), were obtained from streams feeding Lake Michigan and maintained in freshwater tanks at 16 °C until the day of use. All procedures were approved by the Temple University Institutional Animal Care and Use Committee (ACUP# 4610, 26 October 2016). Lampreys were deeply anesthetized by immersion in saturated aqueous benzocaine until motionless to tail pinch, then pinned onto a Sylgard-coated dissecting dish filled with ice-cold lamprey Ringer [14]. The SCs were completely transected at the level of the 5th gill, and the animals allowed to recover in fresh water tanks until use.

### 2.2. Immunohistochemistry

Early axonal regeneration was evaluated at the TX site by applying an antibody against lamprey neurofilaments (NFs) in animals surviving 2 and 4 post-TX. The 1–2 cm lengths of SC rostral and caudal to the TX site at the level of the 5th gill were fixed in 4% paraformaldehyde (PFA) for 3–4 h, washed 3 times in PBS and dehydrated in increasing concentrations of ethanol overnight in a tissue processor and embedded in paraffin. The 10 µm serial horizontal sections (i.e., cut parallel to the dorsal surface) were collected. Then, the sections were deparaffinized in two changes of toluene, and rehydrated in 100%, 95%, 90%, 80%, and 70% ethanol, 5 min each. After three washes in phosphate-buffered saline (PBS) containing 0.2% tween-20, the sections were blocked with 10% fetal bovine serum in PBS for 30 min and incubated with a lamprey NF-specific monoclonal antibody (LCM3 at 1:500 in blocking solution) overnight at 4 °C in a humidified chamber. Next day, after three washes in PBS, the primary antibody was detected by an avidin–biotin complex (ABC) immunohistochemistry kit (Vectastain; Vector Laboratories, Burlingame, CA, USA), using the manufacturer’s protocol. The sections were developed colorimetrically by diaminobenzidine (DAB) chromogen substrate (Thermo Fisher Scientific, Waltham, MA, USA) for 5 min. After 3 washes in PBS, the sections were counterstained for 1 min in hematoxylin solution, then washed in distilled water, dehydrated, cleared, and mounted in Permount.

### 2.3. Retrograde Labeling of Reticulospinal Neurons

To retrogradely label axotomized RS neurons, a pledget of Gelfoam soaked in 5% tetramethylrhodamine-conjugated dextran (DTMR; 10 kDa, Invitrogen, Carlsbad, CA, USA) in tris-HCL buffer (pH 7.4) was placed in a complete fresh TX of the SC at the level of the 5th gill. The animal was euthanized 4 weeks later under benzocaine anesthesia and the brainstem removed and stripped of choroid plexus. After cutting the cerebrotectal commissure along the dorsal midline and extending the obex caudally, the brain was pinned flat on a Sylgard strip [39]. The specimen was fixed in 4% PFA for 2–3 hours, washed 3 times in PBS and stored in 70% ethanol at −20 °C. To retrogradely label RS and propriospinal neurons whose axons had regenerated through the TX at 2, 4, 6 and 13 weeks post-TX, DTMR was applied to a second TX performed at the original TX site (2 weeks), or at 1, 2, 3 or 5 mm (2, 4, 6 or 13 weeks) caudal to the original lesion. The animals were allowed to recover on ice for 2 h before being returned to freshwater tanks. Animals were euthanized 1 week later and processed as above. To test whether re-TX causes regenerated RS neurons to undergo apoptosis, we applied DTMR to a re-TX made 13 weeks after the first TX, allowed the lamprey to survive for an additional 8 weeks, and processed the brain to label apoptotic neurons (see below).

### 2.4. Anterograde Labeling of Degenerating and Regenerating Axons

To investigate axon retraction and regeneration, DTMR was applied to a second TX site at the level of the 2nd gill (~5 mm rostral to the original lesion) at 3 days, 10 days, or 1, 2, 4, 6 or 10 weeks post-TX, as described above, and the animal euthanized 3–7 days later. Longer survival times after labeling were used for animals with more prolonged survivals after the initial TX, to allow the dye to reach the more distant regenerating axons. The brain and SC spanning the original lesion were then removed, fixed, washed and cleared with BABB, as described below. Because glial fibers only begin to invade the lesion site at 10 days post-TX, the lesion is fragile during the first 2 weeks after TX. In order to image the early regenerating axons crossing the lesion, in animals that had survived for 2 weeks post-TX, a length of spinal cord spanning the original TX was removed still attached to the notochord.

### 2.5. Tissue Clearing and Imaging

Brainstems that had been pinned on Sylgard were dehydrated in increasing concentrations of ethanol (70%, 80%, 90%, 95%, and 100% *v/v* in dH_2_O, 20 min each), then carefully freed from the Sylgard strip. To improve visibility, the brainstem and rostral SC were pinned ventral side up on a Sylgard-lined dish filled with 100% EtOH, and the black pigmented connective tissue removed gently from the ventral side of the brain using a Dumont #5 fine-tip forceps (Fine Science Tools Inc., Foster City, CA, USA). Brainstems were transferred to glass vials and clarified in benzyl alcohol/benzyl benzoate (BABB, 1:2 by volume, Thermo Fisher Scientific) solution for 20 min under a laboratory fume hood. The cleared brainstems were whole mounted on glass slides in BABB, which was not allowed to spread beyond the coverslip, and imaged on a widefield fluorescence microscope (Nikon 80i) or on an inverted confocal microscope (Nikon C2) equipped with a motorized stage and NIS-Elements AR software. Z-stack and tiling functions were used to acquire stacks of 5 µm thick slices with 10% overlap between adjacent tiles. After image acquisition, the tissue was stored at 4 °C for future imaging. Under these conditions, fluorescence remained unquenched for at least one month. To prevent signal quenching over longer storage times, the sample was transferred to 100% EtOH and stored in a −20 °C freezer. When working with BABB, gloves were worn, and the use of plastics avoided because the clearing agent is a toxic solvent. Extra attention was paid when using high-power objectives that have very short working distances in order to prevent BABB from dissolving the lens glue.

### 2.6. FLICA Labeling of Apoptotic Neurons

To test whether BABB clearing is compatible with labeling of apoptotic neurons and to observe regenerating RS neurons after extended recovery, DTMR was applied to a SC freshly transected 5 mm caudal to the original TX in a lamprey that had recovered 13 weeks post-TX. After another 8 weeks survival, the brain was removed and processed for labeling of activated caspases with fluorochrome-labeled inhibitors of caspases (FLICA) [19,40]. The brainstem was removed under anesthesia and immediately incubated for 1 h in 150 μL of PBS containing 1 μL of 150× FLICA labeling solution, then washed in full-strength wash buffer in the dark on a nutator, 6 times for 5 min each. All FLICA labeling procedures were carried out at 4 °C. After washes, the brain was pinned flat on a Sylgard strip and fixed in 4% PFA as above. After fixation, the brain was washed in the dark with PBS 3 × 15 min, removed from the Sylgard strip, mounted wet in PBS on a glass slide, observed by wide-field fluorescence (Nikon 80i, Nikon USA; Melville, NY, USA) and photographed. The tissue was then washed, pinned flat on a Sylgard strip, dehydrated, cleared in BABB, and re-imaged.

### 2.7. Correlating Retraction Distances of Axons with Their Diameters

Previous studies had shown that severed axons retract during the first 2 weeks, and the time course of axon retraction has been studied from 2 to 14 days post-TX [18]. However, only axons of large caliber were studied. With the improved image resolution achieved by BABB clearing, we noticed that large axons retract earlier and further than small ones. To correlate axon caliber and the distance of retraction, and to determine the diameters of early regenerating axons descending from the brain, post-TX DTMR was introduced at 2 weeks 5 mm rostral to the original lesion (i.e., less than 2 mm caudal to the spinal–medullary junction). Twenty-four hours later, the SC was removed together with the notochord, pinned straight on a Sylgard strip, fixed, and cleared in BABB. After fluorescent images were captured with widefield microscopy, BABB was removed by rehydration in increasing concentrations of ethanol (70%, 80%, 90%, 95%, and 100%). The sample was dehydrated overnight in a tissue processor and embedded in paraffin. The 10 μm serial transverse sections were collected from a length of SC that spanned from 0.5 mm caudal to the TX site to 1.5 mm rostral to it. IHC with an antibody against lamprey NF was performed as above to label the axons. The sections were photographed under widefield microscopy. The TX site was identified by a widening of the central canal, and by the disordered cellular organization of the SC, with the center of the TX defined as the location of maximum central canal width. Axon retraction was measured as the distance of an axon tip rostral from the center of the TX site. The correlation between retraction distance and axon diameter was documented by measuring the cross-sectional area of the 20 largest axons on a section in every 10th section (every 0.1 mm), using Nikon NIS-Elements AR software (Nikon USA; Melville, NY, USA). The cross-sectional areas were converted to diameters: D = 2 × SQRT(area/3.14).

### 2.8. Cell Counts and Statistical Analysis

To test whether BABB clearing improves the visibility of regenerated RS neurons, in 6 animals that had recovered 13 weeks from SC TX, we applied DTMR to a re-TX made 5 mm caudal to the original TX. After an additional 1 week survival to allow for retrograde transport of the dye, the brains were removed, pinned to the Sylgard and fixed in PFA as above. After three washes in PBS, the brains were mounted in PBS and photographed using a Nikon 80i widefield fluorescence microscope. Then the brains were dehydrated and cleared with BABB as above, and re-imaged. DTMR-positive RS neurons whose axons had regenerated at least 5 mm caudal to the first TX were counted on the captured images from before and after BABB clearing. The differences between the two sets of data were compared and analyzed with InStat software (GraphPad; San Diego, CA, USA). After testing for normality of data, statistical analysis was performed using unpaired two-tailed t-tests with Welch’s correction. The effects of BABB clearing on the appearances of DTMR-positive neurons were determined with regard to total RS neurons, as well as for individual identified neurons. All values were expressed as the mean ± SEM.

To explore the sources of early regenerating axons, in 6 animals at 2 weeks post-TX, DTMR was applied to a re-TX of the original lesion. After an additional 1 week survival, the brain and SC rostral to the TX site were removed, pinned, fixed and cleared as above. The distance from the TX to the obex of the brainstem is approximately 7 mm. This was divided conceptually into 5 segments of approximately 1.4 mm each. The DTMR label identified those neurons whose axons had regenerated as far as the original TX site, and the number of labeled neurons was counted from each segment. The RS neurons in the brain were analyzed separately. Cell counts for different groups of neurons were expressed as the mean ± SEM.

### 2.9. Assessment of the Length of Regenerating Axons

The true lengths of regenerating axons were determined by anterograde labeling from captured confocal images by measuring the distance of each distinguishable axon tip from the TX site. Axons were measured in two animals with 2 weeks survival, one with 4 weeks survival, and one with 10 weeks survival. The TX site was defined by drawing a straight line across the center of the scar. The distance was measured from this line to each axon tip, which was identified by its bulbous enlargement, often with a finger-like distal protrusion. The distance of each axon tip from the TX site was plotted. At longer recovery times, some axons will have regenerated too far to be sure that the anterograde label is filling the distal tip. Therefore, regeneration was assessed in SC wholemounts from animals at 4 (*n* = 4) and 10 weeks (*n* = 8) post-TX by counting anterogradely labeled axons in confocal images (20× objective) as above, at three locations—1 mm rostral (proximal) and 1 and 5 mm caudal to the TX site. The numbers of axons were counted in maximum intensity projection, averaged and graphed.

The numbers of animals used for each experiment are given in Table 1.

## 3. Results

### 3.1. Axons Bridge the Lesion by Two Weeks Post-TX

**Figure 1:**
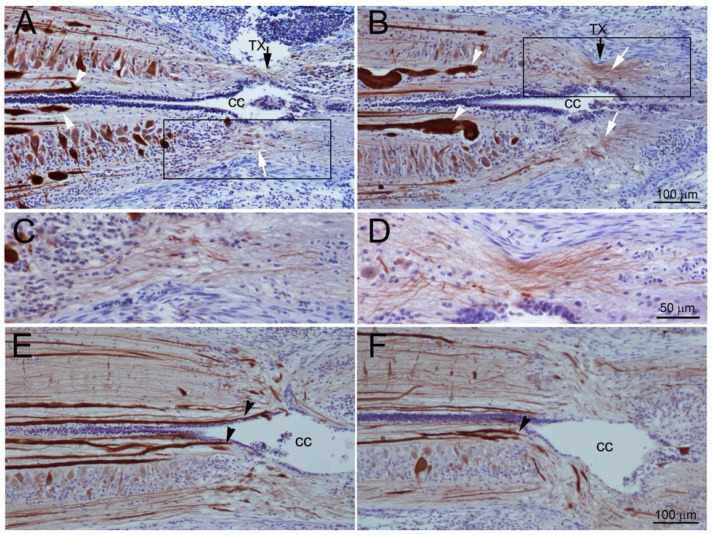
Early regeneration of small-caliber axons across the transection (TX) site. (**A**,**B**), horizontal sections spanning the TX site (black arrows) from two different lampreys at 2 weeks post-TX, stained with an antibody (LCM3) to lamprey NF and conterstained with hematoxylin. Black arrows point to the TX site. The central canal (cc) is expanded and the SC is narrowed, and surrounded by connective tissue. Many fine axons (white arrows) bridge the lesion, but giant reticulospinal (RS) axons have retracted and have not regenerated back to the TX site (white arrowheads). (**C**,**D**), enlarged images from the boxed areas in (**A**,**B**), respectively. (**E**,**F**), horizontal sections from two SCs at 4 weeks post-TX, showing that some giant RS axons have regenerated within the proximal stump and into the lesion (black arrowheads). Thus, there are small axons bridging the lesion by 2 weeks post-TX, while large axons have only just grown back to the injury site by 4 weeks.

Transected axons belonging to the large identified RS neurons retract during the first 2 weeks, then regrow and reach the TX site by 4 weeks [8,9,18]. However, behavioral and electrophysiological studies suggested that regeneration of some axons must be faster than this, and our own previous studies showed that small-caliber neurofilament-containing neurites also enter the injury site from the rostral and caudal stumps as early as 10 days post-TX, following the entrance of glial processes [14]. The origins of these axons, and how long it took for them to bridge the lesion completely was not determined. In the current experiments, immunostaining for neurofilaments confirmed that many small neurites bridged the TX site by 2 weeks post-TX (Figure 1A–D). By 4 weeks, most giant RS axons had reached the TX site (Figure 1E,F).

### 3.2. BABB Clearing Improves Structural Resolution of Retrogradely Labeled Neurons

**Figure 2:**
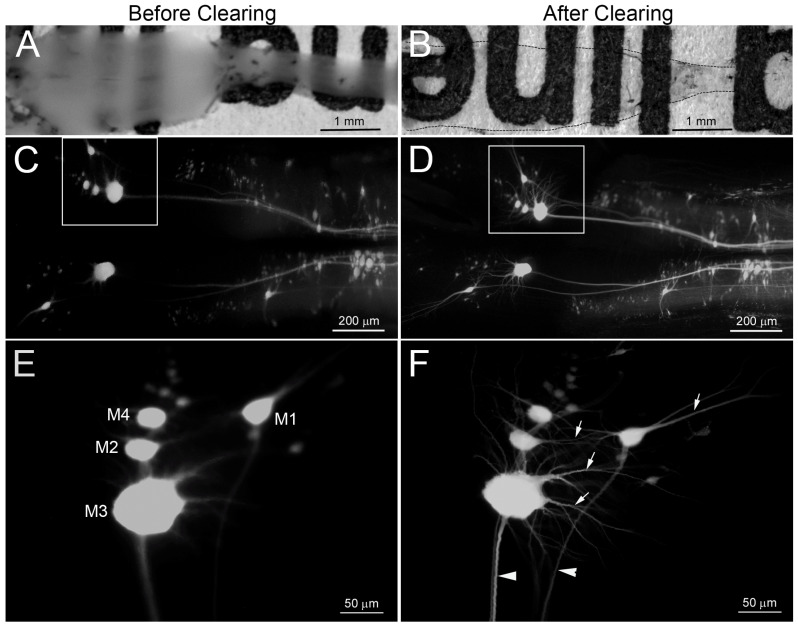
BABB clearing increases the resolution of epifluorescence imaging of RS neurons in brain wholemounts. The SC was transected at the level of the 5th gill and 6 weeks later, surviving RS neurons were labeled retrogradely by application of DTMR to a second TX located at the level of the 2nd gill, 5 mm rostral to the original TX. After allowing one additional week for dye transport, the brainstems were imaged by epifluorescence. (**A**,**C**,**E**) before BABB clearing. (**B**,**D**,**F**) after BABB clearing. (**A**) brightfield image of a lamprey brainstem and SC, which were fixed, washed, placed on a sheet with printed black letters, and photographed with a CCD camera through a stereomicroscope (Olympus, SZX12). Rostral is on the left. (**B**) the same sample is much more transparent after BABB clearing. The specimen is outlined by the dotted line. (**C**–**F**) images captured by a widefield fluorescence microscope (Nikon, 80i) with 4x objective before (**C**) and after (**D**) BABB clearing. (**E**,**F**) images from the boxed areas in (**C**,**D**) enlarged and rotated 90°, so that rostral is now up. The images contain the swollen perikaryal of the identified mesencephalic RS neurons M_1–4_ (note, in unlesioned animals, M_4_ is ordinarily much smaller than M_1–3_—see Figure 4A). Arrows in (**F**) point to dendrites of the M cells; arrowheads indicate descending axons.

**Figure 3:**
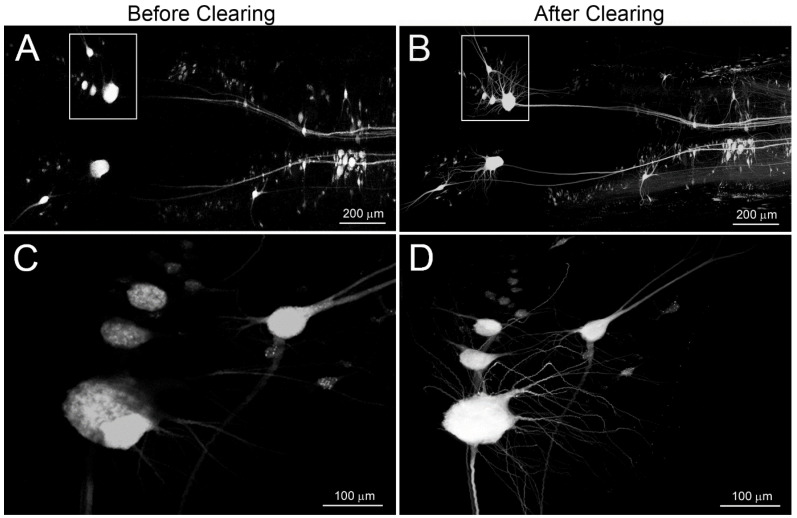
BABB clearing increases resolution of RS neuronal structure in confocal micrographs of brain wholemounts. To compare images between conventional and confocal microscopes, the same sample shown in Figure 1 was imaged with a confocal microscope (Nikon C2) and displayed in the same orientations. (**A**,**C**) before BABB clearing. (**B**,**D**) after BABB clearing. Confocal images were captured in 5 µm optical sections and are shown at maximum intensity projection. (**A**,**B**) were captured under a 10× objective, and (**C**,**D**) under a 20× objective.

The improvement in resolution of neuronal morphology after clearing the tissue with BABB is shown in Figure 2. This shows the effect of prior axotomy on neuronal structure. In a lamprey that survived for 6 weeks after TX at the 5th gill, DTMR was applied to a second TX located 5 mm rostral to the first, to retrogradely label surviving neurons, regardless of whether their axons were regenerating. BABB clearing rendered dehydrated wholemounts prepared from the brain transparent in less than 20 min (Figure 2A vs. Figure 2B), without complicated equipment or expensive reagents. Widefield fluorescence imaging of these wholemounts showed much higher resolution of the same neuronal structures than did conventional fluorescent images from uncleared tissue (Figure 2C vs. Figure 2D). The difference in resolution is more apparent with higher power lenses (Figure 2E vs. Figure 2F). As with other dehydration methods, BABB-treated brains and SCs shrank approximately 30% from their precleared state. Because shrinkage was the same in all three dimensions, however, the tissue retained proportionality. BABB also improved the resolution of neuronal structures imaged by confocal microscopy, which uses a pinhole effect to restrict out of focus background light and enables optical sectioning through tissue up to approximately 125 µm below the surface (Figure 3). Images were optically sectioned at 5 µm and then displayed at maximum intensity projection. Before clearing, lack of transparency prevented light transmission through specimens thicker than 100 μm, which therefore limited the ability of optical sectioning to image deeply enough to capture fine structure (Figure 3A,C). BABB clearing greatly improved resolution of neuronal structures by both increasing transparency and reducing specimen thickness through shrinkage (Figure 3B,D).

**Figure 4:**
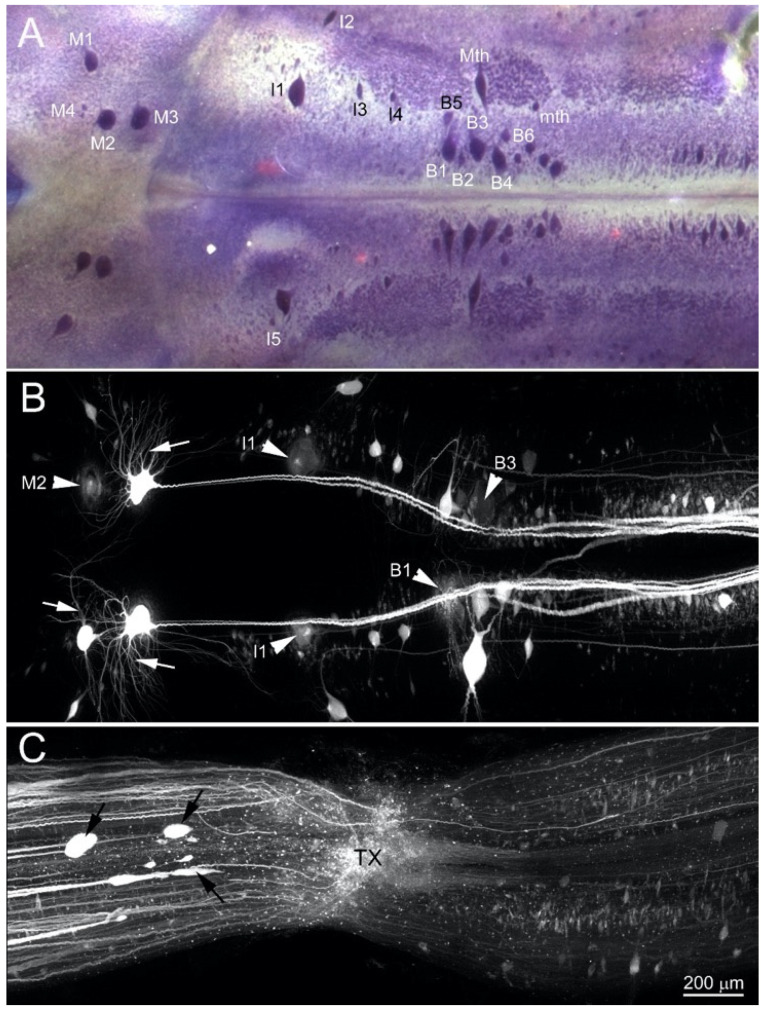
High structural resolution of early degenerative changes in back-labeled neurons and axons after BABB clearing. (**A**) toluidine blue-stained wholemount of a control lamprey brain, showing the large identified RS neurons. (**B**,**C**) RS neurons were labeled retrogradely by application of DTMR to a fresh SC TX at the level of the 5th gill. After 4 weeks recovery, the brain and SC spanning the TX site were removed, fixed and cleared in BABB. (**B**) a wholemount of the brain shows all the RS neurons that project as far as the lesion, including detailed dendritic structures, as seen in focus for neuron M_3_ bilaterally (arrows), as well as numerous small neurons, which could not be seen before clearing. Some swollen RS neurons with faint fluorescent labeling (arrowheads) are undergoing early stage apoptosis, which causes dye to leak out of the cell. (**C**) an image of the SC at the lesion site shows many axons regenerating to, and even beyond, the TX site (rostral is left). White arrows indicate bulbous growing axon tips. The calibration bar in (**C**) is for all three frames.

The neuronal cytoarchitecture of the brainstem and the 18 pairs of identified large/medium RS neurons is illustrated in a wholemount preparation stained with toluidine blue (Figure 4A). Retrograde labeling previously has determined the regenerative probabilities of these [2]. To photograph morphological changes of RS neurons following axotomy, the retrograde label DTMR was applied to the TX site at the 5th gill. Figure 4B,C demonstrate a BABB-cleared brain and SC spanning the TX site at 4 weeks post-TX. Some of the 18 pairs of giant RS neurons survived and are labeled strongly, including their entire dendritic tree (arrows in Figure 4B), but other RS neurons lack normal, smooth membrane edges, and their shape has become indistinct (arrowhead in Figure 4B). The faintness of labeling may have resulted from leakage of the fluorescent dye. These neurons were often surrounded by a blurred fluorescent ring, suggesting that, in addition to dye leakage, these neurons underwent initially swelling, followed by shrinkage. It was often associated with neurons known to have low regenerative probabilities, including B1, B3, M2 and I1 cells (Figure 4B).

By 4 weeks post-TX, many injured axons had regrown back to the TX site (Figure 4C); some had already passed through the scar, and a few formed large bulbous ends before reaching the TX site (black arrows, Figure 4C). It is not clear whether these axons had stopped growing. Because DTMR had been applied to the TX site, background fluorescence and labeled local cells often obscured the regenerating axons.

### 3.3. Application of BABB Clearing after Imaging with FLICA to Assess Regeneration and Apoptotic Signaling in the Same Preparation

**Figure 5:**
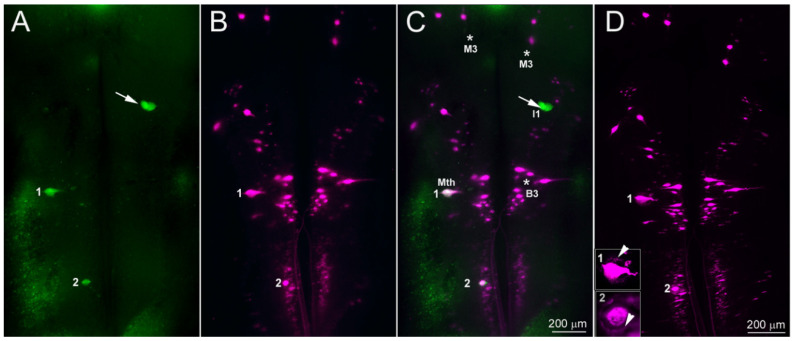
BABB clearing can be applied after FLICA to image regeneration and apoptotic signaling in the same preparation. Thirteen weeks after a TX at the level of the 5th gill, DTMR was applied to a 2nd TX 5 mm caudally. (**A**) after another 8 weeks of recovery, the brain was processed by FLICA (green) to image activated caspases. (**B**) RS neurons whose axons had regenerated at least 5 mm beyond the original TX are labeled red. (**C**) merged (**A**,**B**). A neuron labeled only in green (white arrow in **A**,**C**) is apoptotic due to the original TX. The locations of three identified bad regenerators (the right and left M_3_ and the right B_3_), which are not labeled by either DTMR or FLICA, are indicated by asterisks. These neurons probably are in a late stage of apoptosis or are already dead. Two neurons labeled by both DTMR and FLICA (marked 1 and 2) survived and regenerated their axons after the first TX, but are nevertheless undergoing delayed apoptosis due to the second TX. Number 1 is the left Mauthner neuron, ordinarily a bad regenerator and bad survivor. Number 2 belongs to the left PRRN, whose axons in agregate were reported to have a 31% probability of regenerating [2]. Images in (**A**–**C**) are captured by widefield fluorescence microscopy before BABB clearing. The calibration bar in (**C**) is for all three frames. (**D**) after BABB clearing, the same brainstem shows greatly improved resolution, with approximately 50% more DTMR-labeled neurons visible. The neurons marked 1 and 2 are enlarged in boxes and show pathological morphologies—swelling and impaired membrane integrity (arrowheads).

A frequent use of lamprey CNS in recent years involves detection of apoptotic signaling as a delayed response to axotomy, often using FLICA labeling [40,41]. Thus, it was important to determine whether BABB could be used in conjunction with FLICA. DTMR was applied 5 mm caudal to the original TX at 13 weeks post-TX. Eight weeks later, the tissue was processed by FLICA and imaged by widefield fluorescence microscopy before and after BABB clearing (Figure 5). BABB greatly attenuated the FLICA signal. There were three labeling patterns: 1) DTMR only—neurons whose axons had regenerated (red cells, Figure 5B–D); 2) FLICA only—apoptotic neurons whose axons have not regenerated (arrow, Figure 5A,C); 3) FLICA and DTMR—neurons whose axons regenerated after the 1st TX, but now are apoptotic after the 2nd TX (Figure 5, cells 1 and 2). A few neurons were not labeled by either DTMR or FLICA, e.g., the M3 and B3 neurons indicated by asterisks in Figure 5C. Although they had not become apoptotic despite failing to regenerate, these unlabeled neurons are bad regenerators and most likely were in an advanced stage of apoptosis, or had disappeared altogether. The combination of FLICA and subsequent BABB clearing made it possible to identify apoptotic neurons and obtain a more detailed view of their morphological abnormalities, including swelling and dendritic deformities, than before clearing.

### 3.4. BABB Clearing Increases the Apparent Number of Neurons with Regenerated Axons

**Figure 6:**
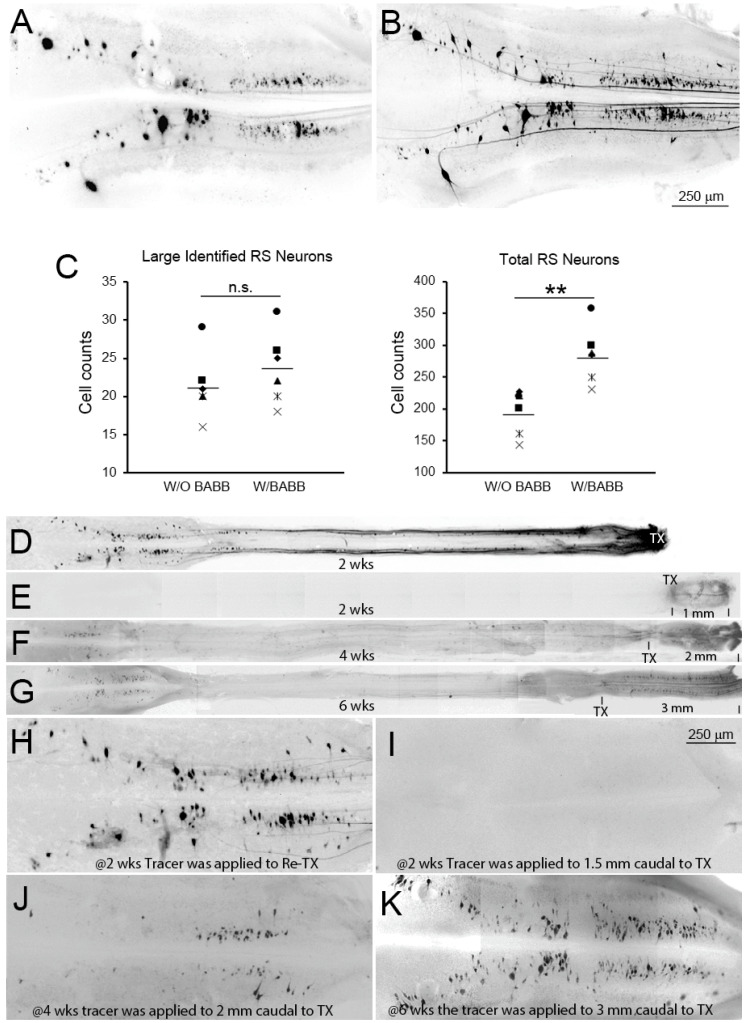
BABB clearing increases the apparent number of neurons with regenerated axons. In 6 animals at 13 wks post-TX, RS neurons with regenerated axons were retrogradely labeled with DTMR from a second TX placed 5 mm caudal to the original lesion. (**A**,**B**) brain wholemounts imaged before (**A**) and after clearing with BABB (**B**), showing an apparent increase in the number of labeled neurons. Image colors are inverted from the original fluorescence for better contrast. (**C**) retrogradely labeled RS neurons were counted in each of the 6 animals before and after clearing. Each of the 6 symbols in the scatter plots represents data from a different animal. The total RS neuron counts increased by 46% after clearing (mean + SEM = 284.8 + 17.9, vs. 195.0 + 14.3; *p* = 0.004), but this was not true for the large identified RS neurons (23.7 + 1.9 vs. 21.3 + 1.7; *p* = 0.391)—ns = not significant; ** *p* < 0.01. (**D**–**G**) DTMR was introduced to a 2nd TX placed at different distances caudal to the original injury site at different recovery times as indicated, i.e., the distances between the 1st and 2nd TX are shown at the lower right hand corner in each frame, and the magnifications are the same. The images were stitched together from low magnification views (objective 2×) to show the entire brainstem and the SC as far as the DTMR application site. (**H**–**K**) enlarged brain images of (**D**–**G**) showing the retrogradely labeled RS neurons. The calibration bar in (**I**) applies to frames (**H**–**K**).

The RS system was examined in BABB-cleared preparations to determine whether conventional fluorescent retrograde tracing in uncleared CNS wholemount preparations results in underestimation of the number of neurons whose axons regenerate after spinal cord TX. That this is true can be appreciated from Figure 5D. In particular, most of the regenerated neurons are much smaller than the large identified RS neurons, and are easily missed. In order to quantify this effect, DTMR was applied to a second TX 5 mm caudal to the original lesion in 6 animals that had recovered 13 weeks post-TX, allowing one week for retrograde transport of the dye. Brain wholemounts were imaged before (Figure 6A) and after clearing with BABB (Figure 6B). Labeled RS neuron counts averaged 46% greater after BABB clearing than before clearing (Figure 6C). The same surgical procedure was performed at different times post-TX, introducing the DTMR label at different distances caudal to the original injury site. In BABB-cleared brains at 2 weeks post-TX, no neurons rostral to the lesion were labeled if the DTMR was applied 1 mm caudal to the first TX (Figure 6E,I). However, when DTMR was applied to a re-TX at the original injury site, many RS neurons were labeled retrogradely in the brain (Figure 6D,H). Thus, at 2 weeks post-TX, regenerating RS axons had passed through the TX site but had not yet penetrated 1 mm into caudal stump. By 4 weeks, small- to medium-sized neurons were labeled retrogradely from 2 mm caudal to the original TX (Figure 6F,J), and by 6 weeks, many more neurons were labeled from 3 mm caudal to the original TX (Figure 6G,K).

### 3.5. Early Regenerating Axons Arise from Small RS Neurons and Small Propriospinal Neurons Near the TX

**Figure 7:**
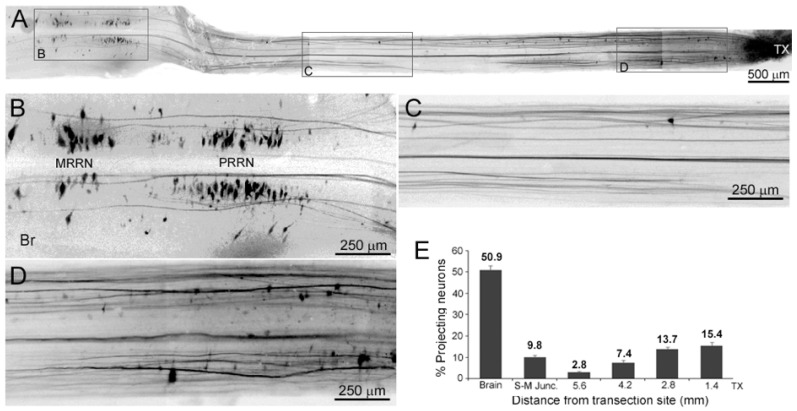
The sources of early regenerating axons. At 2 weeks post-TX, DTMR was applied to a re-TX at the original injury. (**A**) a composite low magnification view showing the entire brainstem and the SC as far as the TX site. The largest number of retrogradely labeled cells are small RS neurons in the brainstem, but some are local neurons near the TX site. (**B**) enlarged images from the boxes in (**A**) show that most early regenerating axons descend from small- to medium-sized RS neurons in the PRRN and MRRN. Very few early regenerating axons derive from the SC ≥ 5 mm rostral to the TX (**C**), but a larger number derive from propriospinal neurons close to the lesion (**D**). (**E**) the distribution of retrogradely labeled neurons at different distances from the TX site (*n* = 6). S-M Junc., spinal–medullary junction.

In 6 animals at 2 weeks post-TX, DTMR was applied to a re-TX at the original injury site, in order to retrogradely label the neurons that gave rise to the earliest-regenerating axons (Figure 7). We expected to find that most of the retrogradely labeled neurons would be local caudally projecting propriospinal interneurons, which are numerous in the lamprey SC [13,17,42,43,44,45]. Although many local interneurons were labeled (Figure 7A), the largest number (51% ± 2.2) of the retrogradely labeled neurons were small- to medium-sized RS neurons in the posterior rhombencephalic reticular nucleus (PRRN) and middle rhombencephalic reticular nucleus (MRRN) (Figure 7B). The number of labeled propriospinal neurons diminished with distance rostral from the lesion (Figure 7E, *n* = 6); very few of the retrogradely labeled neurons were located in segments of SC near the spinal–medullary junction (2.8% ± 0.5, at approximately 5 mm rostral to the TX site at the 5th gill; Figure 7C,E). This pattern was similar in animals labeled at 4 or 6 weeks post-TX (see Figure 6F,G). Thus, in the anterograde tracing experiments described below, the vast majority of early regenerating axons labeled anterogradely by application of DTMR at 5 mm rostral to the original TX (i.e., at the level of the 2nd gill) would have to belong to RS neurons.

### 3.6. Axon Retraction Is Greater in Large Caliber RS Axons than in Narrow Ones

**Figure 8:**
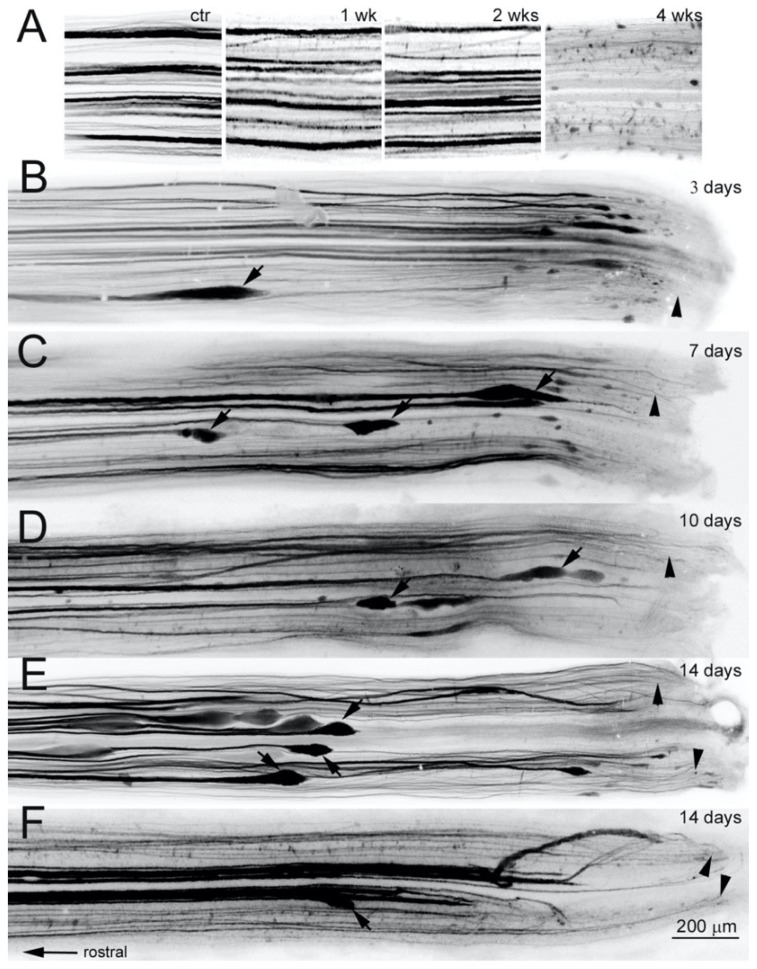
Small caliber axons retracted less than giant axons. (**A**) Wallerian degeneration is slow in the lamprey. (**A**) (ctr), at 3 days post-TX at the 5th gill, retrogradely labeled RS axons a few mm rostral to the TX show no degeneration, and serve as a control. (**A**) (1–4 wks), the SC beginning 5 mm caudal to the TX site at 1, 2 and 4 weeks post-TX, showing anterogradely labeled axons. At 1 and 2 weeks, there were no signs of Wallerian degeneration, whereas by 4 weeks, most giant RS axons had degenerated. Thus, after TX of the SC to introduce a fluorescent label into axons, and allowing up to 1 week for anterograde transport of the dye does not cause distortion of axon structure due to Wallerian degeneration. (**B**–**F**) Axons were anterogradely labeled from a 2nd SC TX near the spinal–medullary junction (~5 mm rostral to original TX) at 3 (**B**), 7 (**C**), 10 (**D**), and 14 (**E**,**F**) days after an initial TX at the 5th gill. Spinal cords were removed, fixed, cleared in BABB, and photographed by wildfield microscopy after allowing 3 days for transport of the dye. The giant RS axons located ventromedially retracted rostrally and developed large bulbous ends (arrows), while small axons remained near the TX site (arrowheads) at all times examined, indicating that small axons retract much less than the large-caliber axons. The TX site is on the right, and rostral is left. Calibration bar = 200 µm for all frames.

It is difficult to determine the distance of retraction of small-caliber axons in uncleared wholemounts, and our previous studies of this phenomenon were restricted to the largest RS axons [18]. This was corrected in the present study by labeling RS axons anterogradely and clearing the SC in BABB, which allowed us to assess initial retraction and early regeneration during the first 2 weeks post-TX. After a predetermined period of recovery from TX, DTMR was inserted into as second TX at the level of the 2nd gill, which allowed us to label caudally-projecting axons anterogradely, while not retrogradely labeling regenerating rostrally projecting axons. Anterograde labeling also avoided introducing additional background fluorescence at or near the lesion site, which occurs in retrograde labeling of the neurons contributing to early regeneration. In order to determine how long we could allow for dye to label axons anterogradely without risking anatomical distortions due to Wallerian degeneration, we labeled axons with fluorescent tracer introduced at the level of the 2nd gill in control animals that had not undergone TX previously, and allowed the animals to recover for 1, 2 or 4 weeks. During the first 2 weeks, there were no obvious signs of Wallerian degeneration at the level of the 5th gill, the location of the usual TX, and 5 mm caudal to the dye application site, when compared to control axons at the same level that had been back labeled from a TX at the level of the 5th gill (Figure 8A). However, the giant RS axons did show signs of Wallerian degeneration at 4 weeks post-TX, the longest time studied. This result confirmed that Wallerian degeneration would not obscure regenerating RS axons near the lesion, which was located 5 mm caudal to the site of dye application.

After an initial TX, anterograde labeling showed that the giant RS axons had retracted a few hundred μm rostrally from the TX and terminated in enlargements, which persist during subsequent regeneration, as described many times by this laboratory and others [7,14,18,32,46,47,48]. The retraction of giant axons began as early as 3 days post-TX (arrow in Figure 8B points to a Mauthner axon). A few large axons retracted much greater distances, even as far as the brainstem during first week after injury as previously described [18,31], and thus were not visible in the length of SC examined. The distance and speed of retraction of those large axons varied, not only because of differences in axon caliber (Figure 8C), but possibly because of other, unknown influences. By comparison, small-caliber axons had retracted very little from the TX site at all observed times (Figure 8B–F, arrowheads).

**Figure 9:**
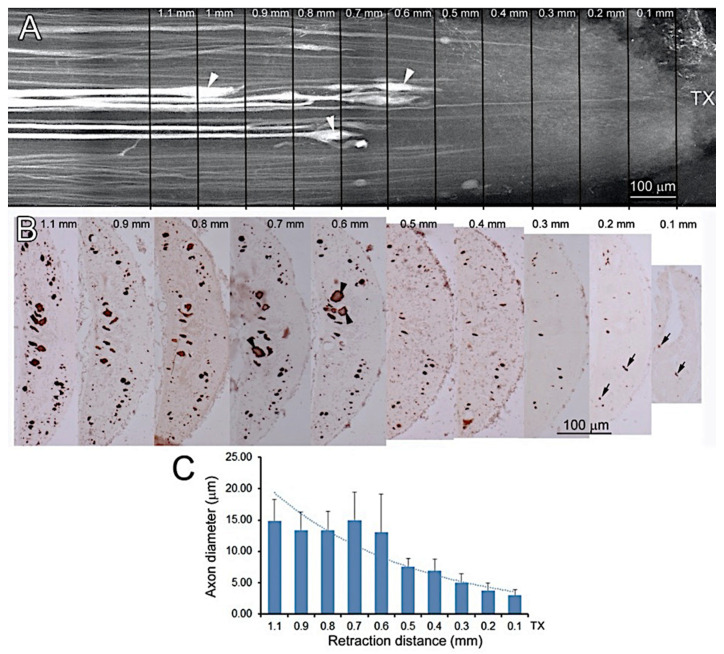
Small axons retract less and regrow through the TX earlier than large axons. At 2 weeks post-TX, axons were anterogradely labeled with DTMR and the SC was removed after only 24 h transport time. After BABB clearing, they were inspected by widefield fluorescence microscopy and processed for paraffin sectioning and IHC. (**A**) Giant RS axons have retracted further and their terminal enlargements (arrowheads) can be seen more rostrally than small axons, even though small axons were not as efficently filled with fluorecent dye. (**B**) serial transverse sections from the same sample as (**A**) stained with the anti-lamprey NF antibody LCM3. The distances from the TX site are marked by lines and numbers. Axons are stained brown. Several small axons remain near or in the TX site (black arrows), but the large ventromedially-located Müller axons (black arrowheads) have retracted rostrally at least 0.5 mm. (**C**) correlation of axon caliber with distance of retraction rostral from the TX site. The diameters of the 20 largest axons on a section were measured in every 10th section from the TX site to 1.1 mm rostrally. The largest-diameter axons have retracted far more than those of small caliber. Note that this procedure excludes some giant axons that have retracted even further than is visible, as well as the smallest axons. Note also that the diameters represent a 30% reduction from the living tissue, due to shrinkage by clearing.

To document the relationship between axon retraction and axon caliber, we studied an additional animal that survived 2 weeks after TX and 24 h after DTMR application just below the spinal–medullary junction. The SC was removed attached to the notochord and photographed after dehydration and clearing (Figure 9A). Serial transverse sections stained for neurofilaments with LCM3 confirmed that small-caliber axons were present near the TX site, and some had grown into the lesion (Figure 9B), but giant axons had retracted at least 0.5 mm rostrally (Figure 9A,B), and had not yet regenerated to the lesion site. The correlation of axon caliber with distance of retraction is shown in Figure 9C. Small caliber axons retracted less than large caliber axons. An apparent ceiling effect, such that retraction did not increase at diameters greater than 15 μm, may be artifactual, since we inspected only a 1.1 mm length of spinal cord, and it is known that some of the largest axons can retract much further, even into the brainstem [18,31], more than 5 mm rostral to the TX at the 5th gill.

### 3.7. Time Course of Axon Regeneration Demonstrated in SC Wholemounts by Anterograde Labeling

**Figure 10:**
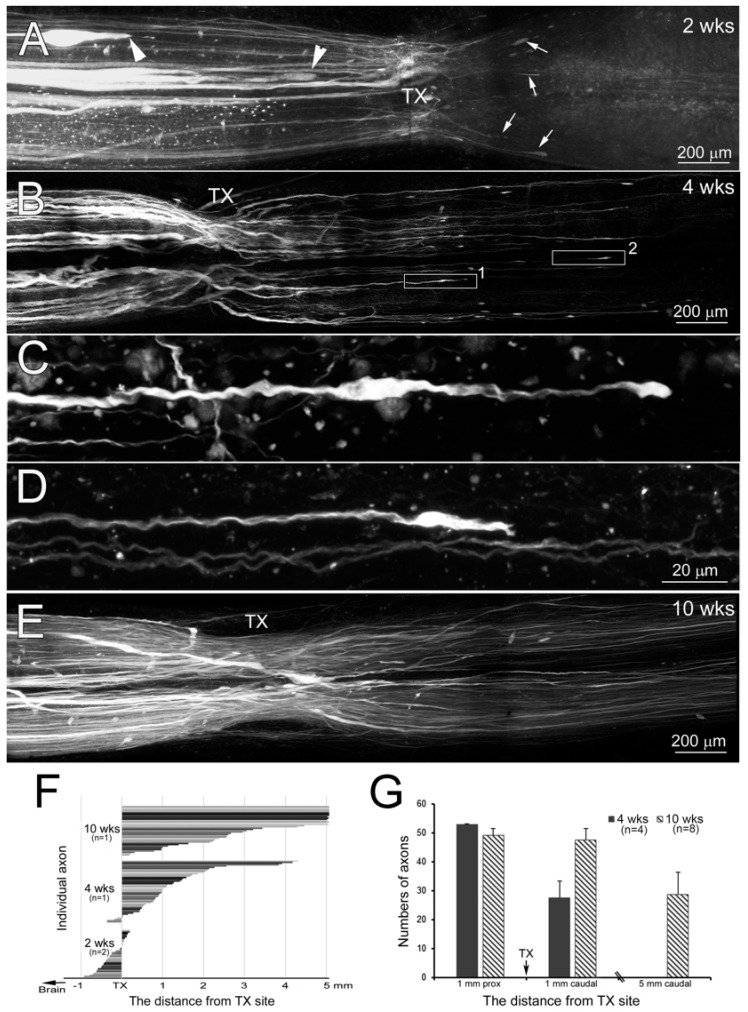
Regenerating axons demonstrated by anterograde labeling and BABB clearing. (**A**,**B**,**E**) maxium intensity projection confocal images of SC spanning the TX site at different recovery times, as indicated. (**A**) some axons have regenerated through lesion into the caudal stump (arrows) as early as 2 weeks post-TX. (**C**,**D**) enlarged images of bulbous axon tips from white boxes 1 and 2 in (**B**), respectively. (**F**) the distance of axon tips to/from the TX site. Each bar represents an individual axon. (**G**) counts of axons from maximum intensity projection confocal images at different distances from the TX, as indicated.

It is difficult to image axons traversing the TX in uncleared wholemounts, not only because of the light-scattering properties of the scar, but also because narrowing of the damaged cord and broadening of the reconstituted central canal greatly increase the packing density of the regenerated axons at the injury site. The regenerated neurites also are slender and their paths within the scar are less straight than their parent axons. Application of fluorescent tracers into the lesion space at the time of TX can retrogradely label the severed axons, but introduces high background fluorescence, which obscures the back-labeled RS axons regenerating across the site of injury. High visibility is particularly critical during the first 4–6 weeks post-TX because intracellular tracer injection studies suggested that the large RS axons of the Müller and Mauthner neurons in the brainstem retract during the first 2 weeks, regenerate into the scar by 4 weeks, and cross the injury by 5–6 weeks [8,9,16,32]. Therefore, we used anterograde labeling together with BABB clearing to examine the time course of RS axon regeneration at 2, 4 and 10 weeks post-TX (Figure 10). The 2-week-old scar tissue is still very fragile, and the SC often separates at the TX site during attempts to remove it from the notochord. In order to image the early regenerating axons passing through the TX scar, 2 weeks post-TX, SCs were removed still attached to the notochord. After fixation, dehydration and BABB clearing, the SCs were imaged by confocal microscopy (Figure 10). A few thin axons had already regrown through lesion into the caudal stump (arrows, Figure 10A). As previously described, the growing tips of large RS axons lacked filopodia, but ended with a terminal enlargement filled with NFs [14,18]. At 2 weeks post-TX, the large axons all terminated rostral to the TX site (arrowheads, Figure 10A). At 4 weeks, many large axons had regrown back to the TX site, and some had grown through it into the caudal stump, although the regenerated fibers were narrower than their parent axons (Figure 10B). As previously described [32,49,50,51], in the caudal stump most axons regenerated on the same side of the midline as their parent axon. However, an occasional axon branched, or looped rostrally, or crossed the midline to extend caudally contralateral to their parent axon (not shown). Numerous thin axons (either from small parent fibers or daughter branches of large parent axons) had regenerated beyond the TX site. Many regenerating large RS axons had distinct bulbous-shaped tips, sometimes ending in a fingerlike projection (Figure 10C,D). By 10 weeks, many, if not most transected RS axons had passed through lesion into the caudal stump (Figure 10E). The distribution of retraction or regeneration at different times post-TX is illustrated in Figure 10F, which plots the distances between the axon tips and TX site in the SC of lampreys that survived for 2–10 weeks. Each bar presents a single axon, and only axons with a recognizable termination were included in the measurements. In two animals at 2 weeks post-TX, most axons had retracted, but a few had already grown into and even beyond the TX. In one animal at 4 weeks post-TX, most anterogradely labeled axons had grown into or beyond the scar, but terminated within 2 mm. None had reached 5 mm beyond the scar. In an animal 10 weeks post-TX, more than half of the labeled of axons had grown more than 5 mm beyond the original TX, although many remained within 5 mm. To test the statistical significance of these findings, the measurements were repeated in four animals at 4 weeks post-TX and in eight animals at 10 weeks (Figure 10G). At 4 weeks, 52% of anterogradely labeled axons reached at least 1 mm caudal to the TX, but less than 1% reached 5 mm. At 10 weeks, 97% of labeled axons reached at least 1 mm beyond the TX, and 51% reached at least 5 mm.

## 4. Discussion

After SC TX in the lamprey, animals become paralyzed below the level of injury, and then gradually recover normal-appearing swimming [52]. The recovery is due to regeneration of axons across the lesion (reviewed in [34]), and because the lamprey contains several pairs of large, individually identified RS neurons, it has been convenient to use them and their large axons in many studies on the mechanisms of CNS axon regeneration. However, the regeneration of these axons is too slow to explain the time course of functional recovery, and in any case, they probably do not serve as descending command neurons for locomotion [53]. The relationships between the qualities of this recovery and axonal regeneration are difficult to assess because they may reflect not only regeneration of axons, but also passive reflexes initiated by movements generated rostral to the TX, as well as variability of restorative processes related to the animal’s age. By some methods, recovery of functional connectivity across the lesion was not observed for the first 5–7 weeks post-TX [32]. However, a combination of electrophysiological and behavioral studies have led most investigators to conclude that lampreys begin to show signs of functional recovery as early as 2–3 weeks after the injury [7,10,11,12], long before the large, individually identified RS neurons regenerate their axons across a complete TX. The present study confirms that as early as two weeks post-TX, the lesion is bridged by smaller axons arising from the neurons of the PRRN and MRRN, as well as from propriospinal neurons located close to the injury, and that this bridging is more robust than previously noted. By combining optical clearing with anterograde and retrograde tracing, these early regenerating projections can be imaged in CNS wholemounts in greater detail and with greater sensitivity than was previously possible without clearing, even using modern brightly fluorescent dyes. This has allowed us to demonstrate that the regenerating RS projections are larger than previously suspected.

### 4.1. BABB Clearing

Compared with paraffin embedding and serial section reconstruction, wholemount fluorescence microscopy has been particularly useful for studying molecular expression after SCI in lamprey by fluorescence in situ hybridization (FISH) and immunohistochemistry. However, application of wholemounts has been somewhat limited by light scattering. In recent years, several optical clearing methods have been proposed to reduce light scattering and facilitate deep imaging in large volumes [54,55,56]. BABB was one of the first clearing methods developed, and is rapid, inexpensive, simple and efficient for multiple label types in many tissues. It can be used with all types of fluorescence microscopes, including conventional widefield, light sheet, confocal and 2 photon.

BABB has been used for developmental studies in lamprey embryos [57,58,59], but has not previously been combined with the wholemount CNS preparations of larval lampreys to study axon regeneration. The absence of myelin in the lamprey CNS has enabled examination of neuronal structure in wholemounts. Nevertheless, in wholemount preparations with neurons injected intracellularly with fluorescent dyes [15,60,61], and in our previous studies using fluorescently labeled retrograde tracers [19,62,63], scattered light from background structures and poor light penetration of thick tissue reduced resolution. By removing these limitations, clearing with BABB greatly improved image quality, revealing greater anatomical detail. The present study does not involve wholemount IHC or ISH, but BABB clearing to enhances imaging with these methods also, and we hope to report on the protocols in a future communication.

Although BABB has been reported to quench fluorescence signals [64,65], we have obtained satisfactory results when we applied BABB to IHC and FISH samples. Unfortunately, FLICA labeling did not survive the use of absolute ethanol during the BABB clearing protocol. However, with FLICA imaging followed by BABB tissue clearing, apoptotic neurons could be identified in the wholemounts.

BABB clearing procedures have been reported to cause tissue shrinkage [66,67], which also occurs with other methods that include dehydration. Our protocol produced an approximately 30% decrease in tissue volume. There was no indication that the shrinkage was different in different dimensions, so it should not produce morphological distortions.

We did not test other clearing methods, but when four tissue clearing methods (BABB, ClearT, Scale, and CLARITY) were quantitatively assessed in mouse brain, using mean free path in optical coherence tomography (OCT) and proton density in magnetic resonance imaging (MRI), BABB was found to be the most effective for both gray and white matter [68]. Similarly, BABB and iDISCO, which uses dibenzyl ether, yielded the best 3D imaging results among 10 clearing solutions, on connective tissue-rich gingiva and skin examined by light-sheet microscopy [55]. In the present study, BABB cleared lamprey CNS completely, rapidly and inexpensively, providing sharp, high-resolution images, even with conventional fluorescence microscopy.

Because BABB is a strong solvent that can dissolve glue [69], it can damage microscope lenses, and must not be allowed to touch the objective lens. However, working distance, rather than light penetration, generally is the limiting factor in imaging cleared tissue, and objectives with large axial travel are essential for accommodating the long working distance of thick wholemount samples. We used only long working distance air objectives (up to 10× in widefield and 20× in confocal microscopes). However, to increase resolution with deep imaging, Leica recently developed a 20× objective lens for confocal microscopy with BABB as the immersion medium.

### 4.2. Early Regenerating RS Axons

A previous IHC study using antibodies against lamprey glial keratins and NF [14] showed that the glial scar was formed to a great degree by longitudinally oriented glial fibers whose cells of origin are in the adjacent SC, and whose glial processes normally are oriented transversely. The longitudinal glial processes entered the lesion by 10 days post-TX, and completely spanned the lesion by 14 days. At that time, small NF-positive processes were described (but not shown) reaching at least to the midline of the scar from both sides, but it was not known whether any of them completely spanned the lesion, and their cells of origin were unknown. We postulated at the time that they might arise from small local interneurons, and might mediate partial functional recovery by relaying signals from un-regenerated RS neurons. The present study aimed more intensely at the early time course of axonal regeneration and confirms that NF-positive fibers span the injury by 2 weeks post-TX, well ahead of the large RS axons. Application of DTMR to the re-transected lesion site 2 weeks post-TX revealed that most of these early regenerating axons derive from small neurons in MRRN and PRRN of the brainstem, the locations where electrical stimulation activates command systems for locomotion [53], both normally and at long times after recovery from SC TX [70]. The validity of this procedure relies on the assumption that the retrograde label does not diffuse in the extracellular fluid and enter neurons remote from the TX across their cell membranes. Evidence for this comes from two sources. First, the DTMR is not membrane permeant, and if applied to the surface of the cord or to the exposed 4th ventricle, does not label cells in uninjured CNS (unpublished). Second, in a previous study [2], we looked for spurious labeling of RS neurons by spread of dye in the CSF. HRP was introduced into a second TX at a time early enough to preclude regeneration but late enough to ensure sealing of injured axon tips [62]. If the HRP was applied after the formation of the glial/ependymal scar (6 days or more), but before any of the large RS axons had regenerated (21 days or less), no large, identified RS neurons were labeled, even if the HRP was applied only 2.5 mm distal to the original TX. At that distance, even smaller RS neurons were not labeled, consistent with the present findings. Thus, diffusion in the extracellular fluid from the spinal cord to the brainstem was ruled out.

In the present study, a smaller number of propriospinal neurons also contributed to the early axon regeneration, and most of these were located close to the original TX. The degree to which these neurons contribute to functional recovery by serving as relays for descending locomotor command systems is not clear, but electrophysiological studies in partial lesion experiments suggested that propriospinal neurons and mechanosensory inputs contribute little to the functional recovery in lampreys, and that the large Müller axons in the ventral columns also do not contribute [70]. Most of the recovery apparently was due to regeneration of axons belonging to smaller locomotor command neurons descending in the lateral columns. Retrograde labeling studies at later recovery times showed regeneration of axons belonging to smaller brainstem neurons located in PRRN and MRRN [2,16]. In the present study, the results of retrograde labeling from the center of the original TX at 2 weeks post-injury, suggest that regeneration of axons belonging to these smaller RS neurons occurs early enough to account for the earliest stages of functional recovery after SCI in the lamprey. We and others have shown that among RS neurons, some have higher probabilities of regenerating their axons than others, but that regenerative abilities of individual neurons and cytoarchitectonic neuron groups are not binary; rather they fall along a continuum [2,6,38]. Moreover, the long-term survival of neurons after axotomy correlates with regenerative ability, as measured at earlier times, and very delayed apoptosis is anticipated by early activation of caspases, many weeks before actual cell death [38,40,71]. The question arises whether this correlation is based on convergence between pathways mediating regeneration and cell survival in the individual neurons, or is a stochastic property emerging in populations of neurons. That so few neurons are double labeled by FLICA and retrogradely transported tracer from caudal to the TX is consistent with our previous findings that those RS neurons that show caspase activity long before they die do not regenerate earlier post-TX, regardless of whether they fall into a category of “good” or “bad” regenerator [72]. In other words, very few neurons regenerate their axons and subsequently die. This does not necessarily mean that the neurons survive because of some consequence of regeneration, such as access to target-derived trophic factors. A previous study showed a correlation between the regenerative abilities of identified RS neurons and their re-expression of the NF protein NF180. This correlation persisted even if regeneration was blocked mechanically [2].

### 4.3. Anterograde Tracing of Descending Axons in CNS Wholemounts

Visibility of back-labeled axons, and continuity near and within a TX, is compromised if tracer is applied directly to the cut ends of the SC at the time of injury. Partly this is because the fluorescent dye is taken up by numerous small cells (including glia) in the region, resulting in high background fluorescence. Although such dye application labels cut axons efficiently, and are useful for retrograde labeling of regenerating neurons, the regeneration of the axons themselves across the lesion may be obscured. Therefore, we used an approach that previously was employed by others in their studies on the effects of cyclic AMP [38] and of repeat TX [37] on axon regeneration in the lamprey SC. We applied tracer to a second TX, 5 mm rostral to the original lesion, i.e., close to the spinal–medullary junction, at 2 weeks post-TX. This allowed us to anterogradely label early regenerating axons descending from the brainstem. Two weeks is much too soon to anticipate rostralward regeneration of axons from neurons located caudal to the original TX. Retrograde labeling showed that such regeneration would have proceeded less than 1.5 mm. Further, because retrograde labeling also showed that very few early regenerating axons belong to propriospinal neurons located 5 mm rostral to the original TX, this anterograde labeling involved RS axons almost exclusively. In order for the tracer to label the regenerating axon tips, in most experiments, we allowed 5–7 days for dye transport. The accuracy of this approach assumes that there will be no distortion due to Wallerian degeneration, which begins within hours in mammalian PNS and is complete by 7–14 days, depending on species, axon caliber (large axons degenerate slowly), and distance from the perikaryon. In the CNS, Wallerian degeneration takes much longer (reviewed in [73]). This distinction may be misleading, because it does not necessarily reflect the time to axonal disintegration, but the time it takes for macrophages (and microglia) to clear myelin debris, and it is the recruitment of macrophages to the injury that is much slower in CNS than PNS [74]. Since lampreys lack myelin, it was necessary to verify that axons remained intact 5 mm or more downstream of the site of axotomy during the time it takes to label their distal regenerating tips. We found that at 1 and 2 weeks post-TX, even small caliber axons remained intact 5 mm downstream of axotomy, whereas by 4 weeks, there was evidence of lost axon integrity. This confirmed earlier findings based on axon labeling with horseradish peroxidase [17]. Thus, we could use anterograde tracing to evaluate both retraction and regeneration of axons in whole-mounted preparations early after TX. The results of this anterograde labeling confirmed that small-caliber spinal-projecting axons from the brainstem regenerated as early as 2 weeks post-TX. Anterograde tracing has been used extensively to document regeneration in the mammalian CNS. Typically, a fluorescent probe is complexed to a carrier such as dextran, and the tracer is injected into the region of the cell body. The intensity of labeling may not be adequate to image small caliber axons in whole-mounted spinal cord, but recently, regeneration of axons in mouse corticospinal tract has been imaged more effectively by injecting an adeno-associated viral vector (AAV) into the cortex to deliver a fluorescent tracer such as green fluorescent protein, and combining this with 3DISCO clearing [75]. A drawback is that this method takes approximately 2 wks to process, much longer than direct injection of dextran-conjugated tracer. Thus far, in our hands, AAV vectors have not worked well on lamprey CNS.

### 4.4. Initial Axon Retraction

After SC TX, proximal axons initially retract, while their distal segments undergo Wallerian degeneration. Whether early regeneration of small axons is due to rapid elongation or to a limited initial axon retraction after TX is uncertain. Transected large RS axons of lampreys retract during the first 2 weeks and then regenerate [8,9,13]. Previous studies revealed that retraction varied among axons, and its extent depended largely on axon size [18,76]. Most small axons retracted less than 0.1 mm, whereas those larger than 20 µm in diameter showed a maximum retraction of up to 1.4 mm at 2 weeks post-TX [18]. A few giant axons retracted as far as the brain. Small axons of the PRRN and MRRN resealed within 15 min of injury, but resealing took as long as 48 h in giant axons, which tend to be bad regenerators [62]. Acceleration of axon resealing reduced axon retraction. These findings suggest that the time required by injured axons to reseal is correlated with the distance of axon retraction, and that faster resealing leads to less retraction. Thus, the small axons that regenerate early post-TX reseal rapidly and retract very short distances. Whether they subsequently grow forward more rapidly than larger axons has not been determined.

### 4.5. Time Course of Regenerating RS Axons

After retraction during the first 2 weeks post-TX, the giant RS axons of lamprey begin to grow, and by 4 weeks, many have grown back as far as the TX site. This growth has been measured within the proximal stump [18,31,47], but the smaller caliber branches regenerating in the distal stump are difficult to track. Axon diameter is also decreased over the first few months following axotomy in mammals [77,78]. The time course of regeneration is difficult to assess by retrograde labeling, particularly because during the first several weeks, very few axons grow far enough to be labeled by tracer applied 5 mm below the original TX [2,79], and it is difficult to predetermine the precise distances of the second TX from the first when these distances are small. However, by anterograde labeling, we were able to examine the early growth of small axons across the TX as early as 2 weeks post-TX, and this regenerated projection grew substantially by 4 weeks. Even at that time, some regenerated axons were seen more than 4 mm caudal to the original TX, but none reached 5 mm. Thus, retrograde labeling from 5 mm caudal to the TX would not detect any regeneration during the first 4 weeks. Because anterograde labeling can be used to study axon retraction and regeneration at the early times of functional recovery (2–4 weeks post-TX), anatomical regeneration can be used to compare results of therapies, and also can greatly shorten the experimental cycle.

## 5. Conclusions

The presence of myelin in most vertebrate species diffuses light, and makes imaging of even fluorescently labeled axons in wholemounts or very thick sections extremely difficult. By contrast, the lamprey lacks myelin and this has allowed much greater resolution of labeled structures. Nevertheless, resolution is far from perfect, and particularly in the region of a SC TX, the disordered paths of regenerating axons, their attenuated diameters, and the light-scattering properties of the many non-neural cells (from blood and possibly connective tissue) that enter the scar make it difficult to follow regenerating axons through the injury site. In the present study, we have combined the higher clarity of BABB with anterograde and retrograde tracing in order to identify the neurons giving rise to axon regeneration as early as two weeks post-TX. Most of these early regenerating axons belong to small RS neurons, but some belong to small local neurons located within 3 mm of the TX. The BABB technique could also be useful for studying structure and regeneration in wholemounts or ultra-thick sections of CNS of other postnatal vertebrates, including mammals.

## Figures and Tables

**Table 1:** Numbers of animals used for each experiment. Wks are weeks after spinal cord transection (TX). The distance of DTMR application caudal to the original TX site is given in parentheses.

Experiments	3 days	1 wk	10 days	2 wks	4 wks	6 wks	10 wks	13 wks
NF immunostaining				2	2			
Retrograde labeling	1				1	1		
Re-TX				6				
Regeneration				1 (1 mm)	1 (2 mm)	1 (3 mm)		6 (5 mm)
Anterograde labeling								
Wallerian degeneration		1		1	1			
Retraction	3	3	4	4				
Regeneration				2	5		9	
